# Free-Chlorine Disinfection as a Selection Pressure on Norovirus

**DOI:** 10.1128/AEM.00244-18

**Published:** 2018-06-18

**Authors:** Andri Taruna Rachmadi, Masaaki Kitajima, Kozo Watanabe, Sakiko Yaegashi, Joeselle Serrana, Arata Nakamura, Toyoko Nakagomi, Osamu Nakagomi, Kazuhiko Katayama, Satoshi Okabe, Daisuke Sano

**Affiliations:** a Division of Environmental Engineering, Hokkaido University, Sapporo, Hokkaido, Japan; b Department of Civil and Environmental Engineering, Tohoku University, Sendai, Miyagi, Japan; c Department of Civil and Environmental Engineering, Ehime University, Matsuyama, Ehime, Japan; d Department of Civil and Environmental Engineering, University of Yamanashi, Kofu, Yamanashi, Japan; e Department of Molecular Microbiology and Immunology, Nagasaki University, Nagasaki, Japan; f Laboratory of Viral Infection I, Department of Infection Control and Immunology, Kitasato Institute for Life Sciences, Kitasato University, Tokyo, Japan; g Graduate School of Environmental Studies, Tohoku University, Sendai, Miyagi, Japan; Centers for Disease Control and Prevention

**Keywords:** free chlorine, genetic drift, mutation, norovirus, selection, susceptibility

## Abstract

Human noroviruses are excreted in feces from infected individuals and included in wastewater. It is critical to remove/inactivate them in wastewater treatment processes, particularly in the disinfection step, before release to aquatic environments. However, the high mutation rates of human noroviruses raise concerns about the emergence of strains that are less susceptible to disinfectants and can survive even after wastewater treatment. This study aimed to demonstrate the strain-dependent susceptibility of norovirus to free chlorine. A population originated from the murine norovirus strain S7-PP3, a surrogate for human noroviruses in environmental testing, was exposed to free chlorine and then propagated in a host cell. This cycle of free chlorine exposure followed by propagation in cells was repeated 10 times, and populations with lower susceptibility to free chlorine were obtained from two independent trials of chlorine exposure cycles. Open reading frame 2 (ORF2) and ORF3 of the murine norovirus genome were analyzed by next-generation sequencing, and a unique nonsynonymous mutation (corresponding to a change from phenylalanine to serine) at nucleotide (nt) 7280 in ORF3, which encodes the minor capsid protein VP2, was found in chlorine-exposed populations from both trials. It was confirmed that all of the clones from the chlorine-treated population had lower susceptibility to free chlorine than those from the control population. These results indicate that exposure to free chlorine and dilution exert different driving forces to form murine norovirus (MNV) quasispecies, and that there is a selective force to form MNV quasispecies under free chlorine exposure.

**IMPORTANCE** This study showed that free chlorine disinfection exerted a selection pressure for murine norovirus (MNV). The strain-dependent viral susceptibility to the disinfectant elucidated in this study highlights the importance of employing less susceptible strains as representative viruses in disinfection tests, because the disinfection rate values obtained from more susceptible strains would be less useful in predicting the virus inactivation efficiency of circulating strains under practical disinfection conditions.

## INTRODUCTION

Norovirus, from the family Caliciviridae, is a nonenveloped virus with an icosahedral shape and a diameter of 38 nm. The norovirus genome consists of single-stranded RNA ∼7.5 kb in length, with three open reading frames (ORFs) (1). Norovirus has been divided into five major genogroups, of which genogroups I (GI), GII, and GIV infect humans (2), and GIII and GV infect bovines and mice, respectively (3, 4). Human noroviruses are a major cause of acute nonbacterial gastroenteritis outbreaks worldwide (5, 6). In the United States, outbreaks caused by human norovirus increased from 1% in 1991 to 12% in 2000 (7). The Centers for Disease Control and Prevention (CDC) reported that approximately 1,753 outbreaks of human norovirus occurred from 2015 to 2016 (8). In Japan, the National Institute of Infectious Diseases (NIID) reported more than 2,400 outbreaks of human norovirus from 2010 to 2017 (9). In England and the Netherlands, there were 4.1 and 4.6 cases, respectively, per 100 person-years of human norovirus incidences in the general population during 1993 to 1996 (10, 11). Human norovirus has accounted for more than 70% of all foodborne pathogens associated with illness and hospitalization in France every year (12). Furthermore, in African and Asian countries, human norovirus is responsible for 22% of diarrhea-related diseases among infants (13).

Human noroviruses are shed in high numbers (more than 10^8^ viruses per gram) from the feces of infected individuals (14) and are relatively stable in the environment for more than 2 weeks (15). The presence of human noroviruses in effluent from wastewater treatment plants (WWTPs) poses a health hazard for humans, and thus the removal/inactivation of human noroviruses at WWTPs is critical to reduce the load of this virus to water environments (16, 17). The final barrier in a wastewater treatment process is generally disinfection, and chlorine-based disinfectants are widely used due to the low cost and efficacy for the inactivation of pathogenic microbes (18, 19). From the viewpoint of a multiple-barrier system, which is the basic concept for the management of human health risks in water usage, it is necessary to determine a representative value of norovirus inactivation efficiency in each treatment process as a form of the log_10_ reduction value (LRV) (20). However, differences in LRVs among norovirus genogroups has been reported. For example, Hata et al. reported mean (± standard deviation [SD]) LRVs of 0.15 (±0.37), 0.49 (±0.14), and 0.38 (±0.72) in a free chlorine treatment for human noroviruses GIV, GII, and GI, respectively (16). Another study reported mean (±SD) LRVs of 1.65 (±1.16) and 2.14 (±0.83) at a WWTP and mean (±SD) LRVs of 2.57 (±1.01) and 2.85 (±0.83) at another WWTP, for noroviruses GI and GII, respectively; both WWTPs employ chlorine disinfection as the final treatment process (17). These results indicate that the susceptibility of human noroviruses to wastewater treatment processes, including free chlorine treatment, varies among genogroups.

The difference in the removal/inactivation efficiency among genotypes/strains of human noroviruses is expected, because genetically diverse strains can emerge due to a high mutation rate. RNA viruses, including human norovirus, tend to undergo higher rates of genome mutation than do DNA-based microorganisms (21–24), which facilitates the diversification of an RNA viral population consisting of mutant spectra, called quasispecies (25). The evolution of viral quasispecies may provide human noroviruses greater opportunities to acquire resistance to disinfectants, because highly resistant or less susceptible strains can be selected through disinfection treatment and survive in the progeny generation. However, the strain-dependent susceptibility of norovirus to disinfection has not been proved so far.

This study thus aims to obtain norovirus with lower susceptibility to free chlorine and determine the genetic changes after free chlorine exposure. In order to achieve this goal, a population originating from murine norovirus (MNV) strain S7-PP3, a surrogate for human noroviruses based on genome similarity and persistency in various conditions (pH, temperature, and disinfectant) compared to those of other surrogates (26), was exposed to free chlorine and then propagated in cell culture. This cycle of free chlorine exposure followed by propagation in cells was repeated 10 times. Then, ORF2 and ORF3 of the murine norovirus genome were analyzed by next-generation sequencing (NGS), and mutations observed in the chlorine-exposed population were compared with the corresponding control (non-chlorine-exposed) populations. Plaque-purified clones containing unique mutations observed only in the chlorine-exposed population were isolated, and the susceptibility to free chlorine, growth rate, and cell binding capability were compared between the mutants and the control clones.

## RESULTS

### Acquisition of MNV populations less susceptible to free chlorine.

**FIG 1:**
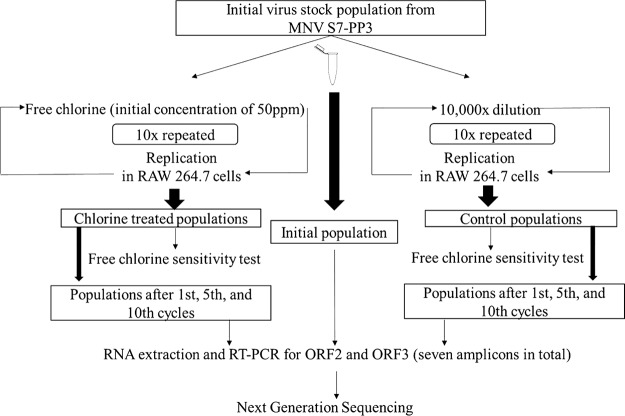
Outline of the cycle experiment.

**FIG 2:**
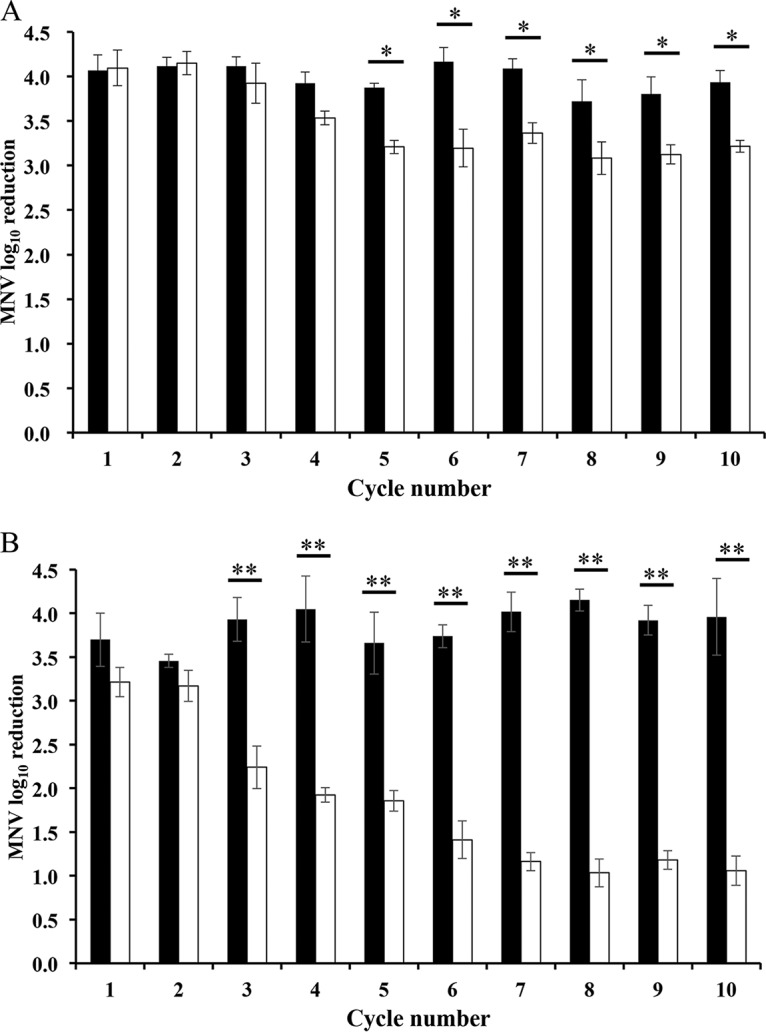
Log_10_ reduction of chlorine-treated population (white bar) and control population (black bar) during the 1st trial (A) and 2nd trial (B) of cycle experiments. *, statistically different at α < 0.05 (Wilcoxon signed-rank test); **, statistically different at α < 0.01 (Wilcoxon signed-rank test).

MNV populations derived from the S7-PP3 strain were repeatedly exposed to free chlorine as shown in Fig. 1. The cycle of free chlorine exposure followed by propagation in RAW 264.7 cells was repeated 10 times (chlorine-treated population). The repeated exposure experiment was conducted twice independently to examine the reproducibility. As a control, the initial MNV population identical with that used for the free chlorine disinfection was diluted 10^4^-fold and propagated in RAW 264.7 cells, and this dilution-propagation cycle was repeated 10 times (control population). The strength of free chlorine disinfection in each cycle was expressed as the product of an initial free chlorine concentration of 50 ppm. This initial concentration level reduced the infectious titer of MNV by 4.11 (±0.14) log (*n* = 3) and 3.21 (±0.15) log (*n* = 3) at the beginnings of the first and second trials, respectively (Fig. 2). At each cycle after the growth in RAW 264.7 cells, the susceptibility to free chlorine was tested for both the chlorine-treated and control populations (Fig. 2). There was no significant difference in the log_10_ reduction value (LRV) in the first four cycles in the first trial of cycle experiments (Fig. 2A) and two cycles in the second trial (Fig. 2B). However, the susceptibility of chlorine-treated populations to free chlorine became gradually lower than that of the control populations, and the difference in LRVs between chlorine-treated and control populations reached 0.71 log in the first trial and 2.74 log in the second trial. These results demonstrate that MNV populations with relatively lower susceptibility to free chlorine were reproducibly obtained by the repeated exposure to free chlorine.

### Single nucleotide polymorphisms (SNPs) in MNV populations.

**FIG 3:**
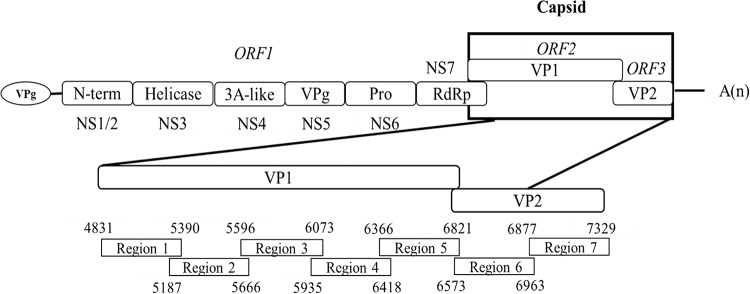
Next-generation sequencing targets in murine norovirus (MNV S7-PP3, accession number: AB435515.1) capsid gene, which consists of ORF2 and ORF3, encoding VP1 and VP2, respectively.

**FIG 4:**
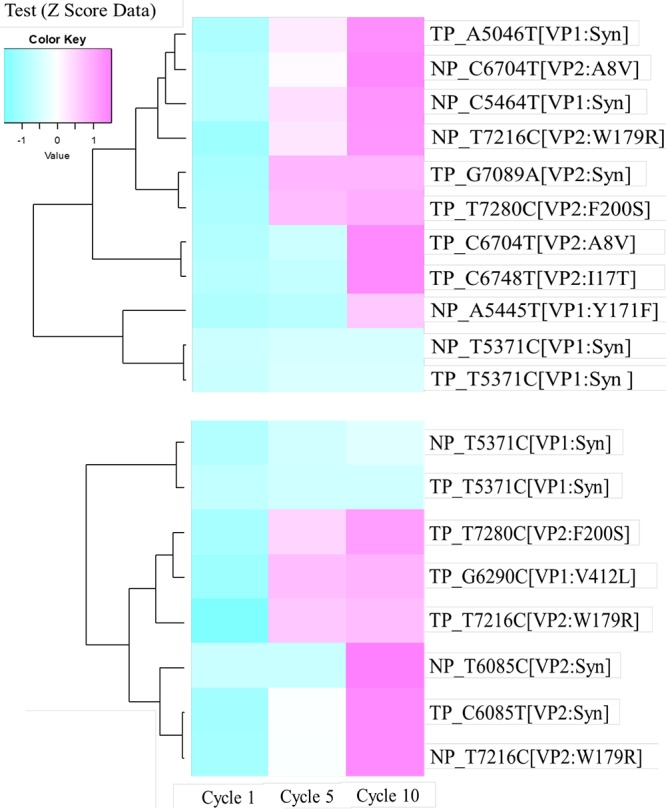
Changes in nucleotides in the 1st, 5th, and 10th cycles in the chlorine-treated and control populations in the first (upper) and second trials (lower). The *Z* score represents the standardized percentage value of a mutation in each cycle.

A next-generation sequencing (NGS) technique was used to investigate the genomic basis for lower chlorine susceptibility of MNV. PCR products of ORF2 and ORF3, divided into seven amplicons (Fig. 3), were obtained from the MNV populations (chlorine-treated and control populations from the first and second trials) in the first, fifth, and tenth cycles, and analyzed by NGS. In total, six nonsynonymous and five synonymous mutations in the first trial and four nonsynonymous and four synonymous mutations in the second trial were found from a 2,252-bp sequence length of ORF2 and ORF3 (Fig. 4). Mutations were more frequently observed in region 7 (nucleotide position of 6877 to 7329; reference strain, MNV S7-PP3; GenBank accession number: AB435515.1) in both trials. Among them, two nonsynonymous and one synonymous mutations were shared by both trials. The shared synonymous mutation was observed at nucleotide (nt) 5371 in ORF2 in both trials with control and chlorine-treated populations. The relative quantity of this mutant among the total reads gradually increased from 61% to 98% in the 10 cycles. One of the two shared nonsynonymous mutations was located at nt 7216 in ORF3, corresponding to a replacement of tryptophan by arginine in the chlorine-treated population from the first trial and in both populations from the second trial. The relative quantity of this nonsynonymous mutation gradually increased from 9% to 99% over the 10 cycles. The other shared nonsynonymous mutation, corresponding to an amino acid substitution of phenylalanine for serine, was located at nt 7280 in ORF3, and it appeared only in the chlorine-treated populations from both trials. The relative quantity for this mutation gradually increased from 1% to 90% and 99% along cycles 1, 5, and 10, respectively. These results indicate that the MNV population with the nonsynonymous mutation at nt 7280 survived better than the wild-type population under the repeated free chlorine exposure.

### Principal-coordinate analysis.

**FIG 5:**
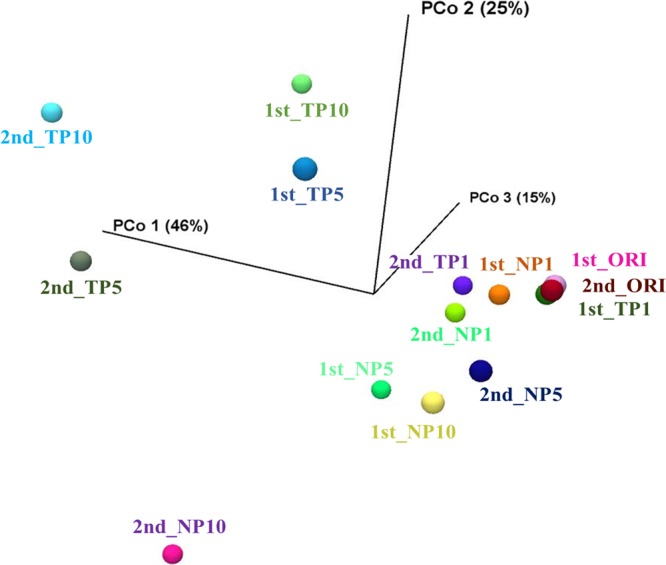
Principal-coordinate analysis used to illustrate similarities and difference in the substitution rate using single-nucleotide polymorphism composition as distance matrices of two populations in the 1st, 5th, and 10th cycles of chlorine-treated (TP) and control populations (NP) in the 1st and 2nd trials. TP1, TP5, and TP10 are chlorine-treated populations in the 1st, 5th, and 10th cycles, respectively. ORI, NP1, NP5, and NP10 are the original population and control populations in the 1st, 5th, and 10th cycles, respectively.

Principal-coordinate analysis (PCoA) was employed to examine the similarities and differences in the substitution rates among the populations, using SNPs as distance matrices in region 7 from the original population (before cycle experiment) and using the chlorine-treated and control populations in the first, fifth, and tenth cycles (Fig. 5). The chlorine-treated and control populations in the first cycle were clustered together close to the original populations. Meanwhile, the chlorine-treated populations in the fifth and tenth cycles were clustered separately from the original and control populations. These results suggest that there was a different driving force to form MNV quasispecies between the exposure to free chlorine and dilution, and that there is a selective force to form MNV quasispecies under the free chlorine exposure. On the other hand, genetic drift caused by dilution may cause different MNV quasispecies in control populations.

### Free chlorine susceptibility of plaque-purified clones.

In order to examine whether SNPs found in the capsid region are associated with lower susceptibility to chlorine, a chlorine sensitivity test was carried out. Six plaque-purified clones were acquired from each population from the first trial. Clones X1, X2, X3, X4, X5, and X6 were obtained from the control population in the tenth cycle, and clones A1, A2, A3, A4, A5, and A6 were obtained from the chlorine-treated population in the tenth cycle. The genome sequence of region 7 was also determined by the Sanger sequencing method, and it was found that all clones from the chlorine-treated population (A1 to A6) had the nonsynonymous mutation at nt 7280 (see Fig. S1 in the supplemental material).

**FIG 6:**
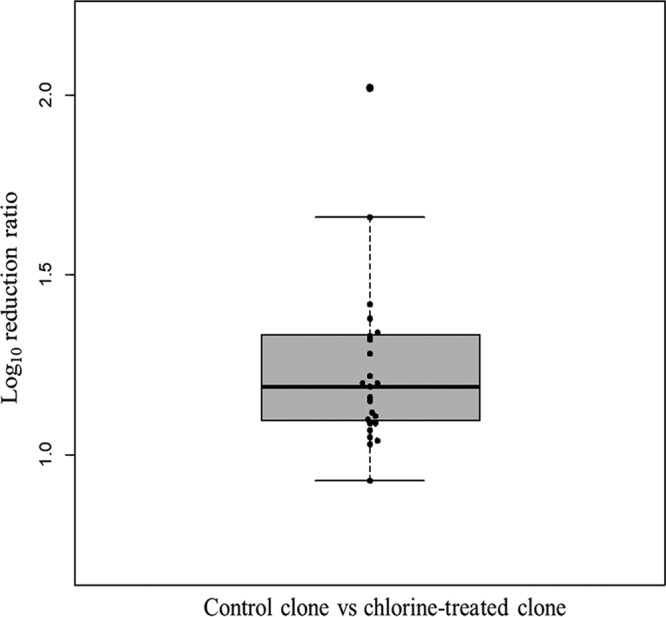
Log_10_ reduction ratio of clones from the control population (X clones) to those from the chlorine-treated population (A clones). The chlorine sensitivity test was conducted for nine clone combinations (X1/A1, X1/A2, X1/A3, X2/A1, X2/A2, X2/A3, X3/A1, X3/A2, X3/A3) with initial concentration of 2 ppm, and 1 min incubation. The test was repeated three times for each combination, and there are 27 dots in total.

The free chlorine sensitivity of the plaque-purified clones was evaluated to investigate whether the nonsynonymous mutation (T7280C[VP2:F200S]) would affect the susceptibility of MNV to free chlorine. An X clone was paired with an A clone, and nine pairs were selected randomly (A1 versus X1, A1 versus X2, A1 versus X3, A2 versus X1, A2 versus X2, A2 versus X3, A3 versus X1, A3 versus X2, and A3 versus X3). Then, the log_10_ reduction ratio after free chlorine exposure with an initial concentration of 2 ppm and 1 min contact time was obtained 3 times for each pair, providing the results represented by the 27 dots in Fig. 6. The average of ratio values was 1.24, and the minimum and maximum ratio values were 0.93 and 2.02, respectively. These ratio values were significantly greater than 1.0 (one-sample Wilcoxon signed-rank test, *P* = 5.22 ×10^−8^), which means that the plaque-purified clones from the chlorine-treated population had significantly lower susceptibility to free chlorine than those from control populations.

### Replicative fitness of plaque-purified clones.

A one-step growth curve experiment was conducted to investigate whether clones isolated from the chlorine-treated population after the tenth cycle had higher replicative fitness compared to clones from the control population. The growth rate and doubling time, calculated from a one-step growth curve of each plaque-purified clone from the control and chlorine-treated populations (six clones in total), ranged from 0.76 to 0.83 infectious virus particles/hour and 0.84 to 0.91 h, respectively (see Table S1 in the supplemental material). There were no statistically significant differences in the growth rate and doubling time between the clones from the chlorine-treated and control populations, which were tested using the Kruskal-Wallis test (*P* = 0.07 for growth rate and *P* = 0.19 for doubling time). The selection coefficient (*s*) (equation 4) of plaque-purified clones from the chlorine-treated and control populations was calculated to compare the relative replication fitness of the clones. There was no statistically significant difference in the selection coefficients (*s*) among tested clones, as determined by the Kruskal-Wallis test (*P* = 0.06; see Table S2 in the supplemental material). The positive value of the selection coefficient (>0) suggested that the clones from the chlorine-treated population had a slightly lower fitness value compared to the clones from the control population. However, statistical tests showed that s values were not higher than 0 (Wilcoxon signed-rank test, *P* = 0.002, α = 0.05), indicating no significance difference in the replicative fitness among the clones.

### Binding efficiency to cell surface.

The binding efficiency of plaque-purified clones with and without the nonsynonymous mutation (T7280C[VP2:F200S]) to RAW 264.7 cell surface was evaluated. The number of viral particles bound to the host cell surface was determined by RT-qPCR, and we found that the average values of the bound MNV count divided by the spiked MNV count were −2.24 and −1.69 log_10_ for clones from the chlorine-treated (with T7280C[VP2:F200S]) and control populations, respectively (see Fig. S2 in the supplemental material). Statistical tests showed that the ratio of bound viral gene copy number to added copy number was not significantly different between mutants (T7280C[VP2:F200S]) and the control clones (Wilcoxon rank sum test, *P* = 0.98), which indicated that all clones exhibited a similar ability to bind to the host cells.

## DISCUSSION

We have demonstrated the viral strain dependency of free chlorine susceptibility. The NGS results showed that the ratio of a nonsynonymous mutation in ORF3, which encodes the minor capsid protein VP2, gradually increased when the MNV population was repeatedly treated with free chlorine. The plaque-purified clones from the chlorine-treated population, which shared the same nonsynonymous mutation at nucleotide (nt) number 7280, had significantly lower susceptibility to free chlorine than those from the control populations without the nonsynonymous mutation. The growth rate and binding efficiency to the host cell surface of plaque-purified clones from the chlorine-treated population were not significantly different from those of the control population.

Viral quasispecies evolution is influenced by population genetics factors, including mutation, selection, and genetic drift (27). The MNV S7 strain was known to exhibit higher genetic diversity compared to MNV-1, which indicates the coexistence of multiple haplotypes in a viral isolate (28). In both the chlorine-treated and control populations, a larger number of nucleotide changes was observed in ORF3 than in ORF2 (Fig. 4). A previous study investigating *in vitro* replication of MNV in RAW 264.7 cells also reported a higher mutation rate in ORF3 (53.13 × 10^−5^ mutation/site/day) compared to ORF2 (12.89 × 10^−5^ mutation/site/day) (29). The reason why the mutation rate in ORF3 of MNV is higher than that in ORF2 remains unclear, but VP2 may have a lower structural constraint than VP1, which could explain the accumulation of mutations in ORF3. The three-dimensional (3D) structure and localization of VP2 in a virion need to be determined to understand the mechanism of the higher mutation rate in ORF3 than in ORF2.

Larger numbers of synonymous and nonsynonymous mutations were observed in the chlorine-treated populations than in the control populations (Fig. 4), and the direction of evolution of the chlorine-treated populations was different from that of the control populations (Fig. 5). These results indicate that free chlorine treatment has an effect on norovirus evolution distinct from that of genetic drift. Through free chlorine selection, strains that are less susceptible to free chlorine may be selected. The frequencies of these strains increased with the numbers of treatment cycles. These less susceptible strains may have already existed in the original population in a lower proportion. However, the larger number of mutations found in the treated populations underpins another possibility, namely, that the strains were newly developed via mutation during the treatments.

Some synonymous and nonsynonymous mutations were observed in the control populations from both trials (Fig. 4). The control population was not exposed to free chlorine, but the culture was diluted 10,000-fold before replication in host cells at each cycle. This dilution at each cycle introduces a bottleneck effect to the MNV population, in which only a small subset of the population is allowed to produce progeny in the host cells. In other words, the control population reflects only the evolutionary forces of mutation and genetic drift, eliminating the effect of selection under chorine treatment. The PCoA pattern found in control populations showed a smaller evolutionary shift from the original population compared to the chlorine-treated populations, except for the population at the tenth cycles of the second trial (Fig. 5). Mutations of control populations may be balanced by genetic drift, which tends to remove mutants and constrain population divergence. A previous study also showed that the repetitious passage of MNV S7 at low multiplicity of infection (MOI), in which a genetic bottleneck is emphasized, resulted in a convergence in the frequency of haplotypes (28).

The acquisition of less susceptible populations after repeated exposure to chlorine-based disinfectants was also observed in different species of RNA virus. For example, a chlorine dioxide-exposed population of bacteriophage MS2 with nonsynonymous mutations of A467T and A1443G showed lower susceptibility to chlorine dioxide (30). In the case of echovirus 11, a chlorine dioxide-treated population included nonsynonymous mutations of G1373C, A2397T, and C3101A, and this population showed less sensitivity to chlorine dioxide than the original population (31). These previous studies suggest that nonsynonymous mutations appearing after the repeated exposure to the chlorine disinfectant were linked to the improvement of RNA viruses to bind with the host cell surface due to the amino acid substitution of chlorine-labile amino acids for chlorine-stabile ones (30, 31). The echovirus study concluded that the lower susceptibility to chlorine dioxide was caused by the acquisition ability of the virus to utilize alternative cell receptors to attach to the host cell surface, due to nonsynonymous mutations in the capsid region (31).

The ability of MNV to bind to glycolipid and glycoprotein attachment receptors on the host cell surface may be strain dependent, since a mutant with only one amino acid change from a wild type was able to exhibit distinct changes in tissue tropism (32). Some MNV lineages, such as MNV-1, WU11, and S99, bind *ex vivo* to microphages through a terminal sialic acid on the ganglioside GD1a (33), whereas other strains, such as CR3, can bind to another glycan (32), which means that the lower susceptibility of MNV populations observed in the present study can also be explained by the change in the binding efficiency to the host cell surface. However, the nonsynonymous mutation that appeared only in the chlorine-treated populations (T7280C) was located in ORF3, encoding VP2, which does not have a role for binding to the host cell surface, in contrast to the VP1 protein (34, 35). Our results showed that all plaque-purified strains with T7280C[VP2:F200S] exhibited lower susceptibility to free chlorine compared to that of control strains, but there were no statistical differences in the replicative fitness (Table S2) and binding ability (Fig. S2) between them, which suggests that the lower susceptibility to free chlorine was not an apparent outcome caused by higher binding and replication efficiencies.

One possible explanation for the lower susceptibility to free chlorine is that the VP2 protein increases the stability of the capsid by protecting VP1 proteins from disassembly due to free chlorine exposure (36–38). A previous study reported that the yield of human norovirus-like particles (VLP) was low if the ORF3 was not included in the recombinant genome, which suggests that VP2 has a pivotal role in forming a virion of human norovirus (39). Although an analogical inference between the capsid structure of human and murine noroviruses is not easy because the localization of VP2 has so far only been analyzed for Norwalk virus (40), the contribution of T7280C[VP2:F200S] to the structural stability of the MNV virion may be proved by information about the 3D location of MNV VP2 in the future.

It must be noted that there was a clear difference in the susceptibility to free chlorine between the first and second trials of the chlorine-treated population (Fig. 2). This difference cannot be explained by the mutation at nt 7280, because clones from both populations share this mutation. A possible explanation is that the other mutations also play roles in chlorine susceptibility. For example, a nonsynonymous mutation (G6290C[VP1:V412L]) was observed only for the chlorine-treated population from the 2nd trial. There is also a possibility that mutations in ORF1, which encodes nonstructural proteins, affect the free chlorine susceptibility, although this region was not analyzed in the present study. The large variation in the log_10_ reduction ratio in Fig. 6 can be also explained by the presence of multiple mutations responsible for the free chlorine susceptibility. A synergistic effect of multiple mutations in the MNV genome on the free chlorine susceptibility needs to be investigated in further studies.

This study demonstrated that free chlorine disinfection acted as a selection pressure on an MNV population. If this is also the case for human noroviruses, it is important to use less susceptible (stronger) strains in a water disinfection test for estimating the virus inactivation rate; otherwise, the disinfection efficiency in an actual setting does not reach the expected level and an infection risk is posed even with disinfected water. The strain-dependent susceptibility of RNA viruses to a variety of disinfection technologies, including ozonation and UV light irradiation, and the possibilities of cross-resistance with other disinfectant and or antivirals which have been described previously (41, 42) need to be further investigated to achieve safer usage of water that may be contaminated by pathogenic viruses.

## MATERIALS AND METHODS

### MNV isolates and cell lines for chlorine cycle experiment.

The MNV S7-PP3 strain, which is genetically close to MNV3 (43), was obtained from Yukinobu Tohya, Nihon University, Japan. Both MNV S7 strains and a prototype MNV-1 strain have been used as foodborne pathogen models for human noroviruses. MNV S7 strains have been widely used by Japanese and U.S. researchers as a free chlorine inactivation model (44), in a test for a plant-based antiviral substance (45), and in an investigation of functional receptor for MNV (34). MNV S7-PP3 cells were propagated, enumerated, and purified according to the published protocols (46). Viral stocks were stored at −81°C before use. RAW 264.7 cells (ATCC TIB-71) were used as host cells and cultured in modified Dulbecco's modified Eagle's medium (DMEM) containing 10% (vol/vol) fetal bovine serum (FBS), 0.075% NaHCO_3_, 2 mM l-glutamine, 10 mM nonessential amino acids, 100 mg/ml penicillin, and 100 U/ml streptomycin. Cells were cultured at 37°C and 5% CO_2_.

### Chlorine cycle experiment.

To investigate the lowering of susceptibility of norovirus to free chlorine, MNV S7-PP3 was treated with free chlorine repeatedly 10 times (Fig. 1). This cycle experiment was conducted twice. The free chlorine solution was prepared by mixing sodium hypochlorite solution (Wako, Osaka, Japan) with phosphate-buffered saline (PBS) solution with an initial chlorine concentration of 50 ppm, measured using a chlorine comparator DPD (*N*,*N*-diethyl-*p*-phenylenediamine) method (Shibata, Saitama, Japan). Surviving virus from the first chlorine cycle was regrown in RAW 264.7 cells and repeated for another nine cycles. As a control treatment, the MNV S7-PP3 population was subjected to repetitious growth in RAW 264.7 cells for 10 cycles without chlorine exposure. As a control, the initial MNV population identical with that used for the free chlorine disinfection was diluted 10^4^-fold and propagated in RAW 264.7 cells, and this dilution-propagation cycle was repeated 10 times to obtain a control population. Chlorine susceptibility for both control and chlorine-treated populations in each cycle was determined by measuring log_10_ reduction of exposure to free chlorine.

### Next-generation sequencing (NGS).

To examine genetic changes in MNV S7-PP3 populations due to the free chlorine intervention, next-generation sequencing (NGS) was employed for both the first and second trials of the cycle experiment. The sequencing target was the capsid region of MNV, since it is known that free chlorine cause damages to the viral capsid (47). The MNV S7-PP3 capsid gene (2,252 bp in length) proteins were divided into seven regions (Fig. 3). Primers were designed to cover all seven regions with an overlapping sequence (Table 1). The original population, the fifth and tenth cycles of the control, and the free chlorine-treated populations were picked for NGS. The process of amplicon preparation involves RNA extraction, reverse transcription-PCR (RT-PCR), PCR targeting ORF2 and ORF3, gel electrophoresis to check the length of the PCR products, and PCR product purification from the gel. Reverse transcription-PCR, PCR, and PCR product purification were performed following the PrimeScript Perfect real-time RT reagent (TaKaRa Bio, Shiga, Japan), the Phusion high fidelity PCR kit (New England BioLabs, Ipswich, MA), and the Fast Gene gel/PCR purification kit (Nippon Genetics, Tokyo, Japan) manufacturers' protocols, respectively. The PCR temperature profile is shown in Table S3 in the supplemental material.

**TABLE 1:** Primers used for sequencing of MNV ORF2 and ORF3

Primer	Sequence (5′–3′)^*a*^	Location (nucleotide position)^*b*^
MNV-ORF2-AN1-F-Pho	[PHO]-CACTCCCAGGACATGCTCAG	4831–4850
MNV-ORF2-AN1-R-Pho	[PHO]-CCGAGGGCCAGATCAAACAA	5290–5390
MNV-ORF2-AN2-F-Pho	[PHO]-CGCCGGGCAAATCAATCAAA	5187–5206
MNV-ORF2-AN2-R-Pho	[PHO]-CGGCCAGAGACCACAAAAGA	5647–5666
MNV-ORF2-AN3-F-Pho	[PHO]-CTGGTTTGCATGCTGTACACG	5596–5616
MNV-ORF2-AN3-R-Pho	[PHO]-GCACCTCGATCTCTAGTTGTCC	6052–6073
MNV-ORF2-AN4-F-Pho	[PHO]-GCTTACGAGTTCCAGTCCGG	5935–5954
MNV-ORF2-AN4-R-Pho	[PHO]-GTGGAAGGGCACAGTCGATG	6399–6418
MNV-ORF2-AN5-F-Pho	[PHO]-TCAGATTGACAGCACTGACGC	6366–6386
MNV-ORF2-AN5-R-Pho	[PHO]-CGTTGCAAGCAGGGAAGAATTG	6800–6821
MNV-ORF3-AN1-F-Pho	[PHO]-ACTCACCTTCCCGACTGATG	6573–6592
MNV-ORF3-AN1-R-Pho	[PHO]-TGTTGATGGCATTCTCCTGGG	6943–6963
MNV-ORF3-AN2-F-Pho	[PHO]-TCCAAACCAACTCTTTCAAGCA	6877–6898
MNV-ORF3-AN2-R-Pho	[PHO]-CACAAAAGGTTTCTCTTCCAAC	7308–7329

a [PHO], phosphorylated primer.

b Nucleotide location was determined using MNVS7-PP3 references (GenBank accession number AB435515.1).

In order to prepare MiSeq libraries for the first trial of the cycle experiment, each PCR product was ligated to different Y type adapters, which consist of i5 (D501 to 507) and i7 (D701 to 707) oligomers (48). First, 49 types of the Y adapters were prepared by annealing each i5 and i7 oligomer. Annealing mixtures contained 6 μl of one of the i5 oligomers (50 μM), 6 μl of one of the i7 oligomers phosphorylated in 5′-end (50 μM), and 1.35 μl of an annealing buffer (0.06 M Tris-HCl, 0.01 M EDTA, 0.025 M NaCl). The annealing mixture was incubated on a T100 thermal cycler (Bio-Rad, Hercules, CA) with the following steps: 5 min at 95°C, 140 ×30 s with the temperature decreased by 0.5°C per each cycle, 40 min at 25°C, and storage at 12°C.

The PCR products were adenylated at the 3′-end in a 50-μl reaction volume with PCR products at 0.15 to 1.5 pmol, 5 μl Ex Taq buffer (TaKaRa), 3 μl MgCl_2_ (TaKaRa) (25 mM), 1 μl dATP (Promega, Madison, WI) (10 mM), 2.5 U Ex Taq (TaKaRa), and PCR-grade water up to 50 μl. An adenylation reaction was performed at 72°C for one hour by a T100 thermal cycler (Bio-Rad). Adenylated products were purified by the QIAquick PCR purification kit (Qiagen, Hilden, Germany), and DNA concentration was measured by the Quantus fluorometer using the QuantiFluor double-stranded DNA (dsDNA) system (Promega). These adenylated products were ligated to a different Y adapter as soon as possible after adenylation. The reaction mixture consisted of 50 μl, with 5 μl ligation buffer (TaKaRa), 1 μl T4 DNA ligase (TaKaRa), 40 μl each adenylated product, one of the Y-type adapters at 100× the molar amount of the adenylated product, and PCR-grade water up to 50 μl. Ligation reactions were performed for 19 h at 16°C and 2 min at 65°C by a T100 thermal cycler (Bio-Rad). Ligation products were purified by the Agencourt AMPureXP system (Beckman Coulter, Brea, CA), and DNA concentration was measured by the Quantus fluorometer using the QuantiFluor dsDNA System (Promega).

To enrich the DNA libraries, a second PCR was performed in a 10-μl reaction mixture with Protocol Phusion high-fidelity PCR master mix with 5 μl HF buffer (New England BioLabs), 0.5 μl Kapa Illumina primer P1 (5′-AAT GAT ACG GCG ACC GA-3′) (10 μM), 0.5 μl Illumina primer P2 (5′-CAA GCA GAA GAC GGC ATA CGA-3′) (10 μM), 3 μl each ligated DNA, and PCR-grade water up to 10 μl. PCR was performed under the following temperature profiles: 30 s at 94°C, 15 cycles of 10 s at 94°C, 30 s at 60°C, 1 min at 72°C, a final extension step of 5 min at 72°C, and storage at 4°C. PCR products were purified using the Agencourt AMPureXP system (Beckman Coulter).

A quality check for all libraries was performed by the Bioanalyzer (Agilent), using the High Sensitivity DNA kit (Agilent). To quantitate the concentration of the library DNA, real-time PCR was performed in a 10-μl reaction volume with 5 μl Kapa SYBR Fast quantitative PCR (qPCR) master mix (Kapa), 0.2 μl Illumina P1 primer (10 μM), 0.2 μl Illumina P2 primer (10 μM), 2 μl 1,000×-diluted library DNA or DNA quantitative standards (Kapa), and 2.6 μl PCR-grade water. Each quantification was performed in triplicate. PCR cycle conditions followed the protocols of the Kapa library quantification kits. Forty-nine libraries were pooled in equal molar amounts. Because the concentration of the pooled library was too low to run MiSeq, this pooled library was concentrated using the Agencourt AMPureXP (Beckman Coulter). The pooled library was again quantified according to the same conditions as the real-time PCR and was adjusted to 2 nM. PhiX control (30%; Illumina) was spiked into the library. Finally, 600 μl of 6 pM denatured library was prepared, and 300-bp paired-end sequencing was carried out by the Advanced Research Support Center at Ehime University using the MiSeq platform (Illumina).

We used a library preparation kit in the second trial that was not used in the 1st trial. The kit saved a lot of time in the library preparation. The amplicon library was prepared using the TruSeq DNA PCR-Free LT library preparation kit (catalog no. FC-121-3001 and FC-121-3003; Illumina, Inc.) on PCR products amplified with phosphorylated primers. Steps from the kit's protocol guide were followed from the adenylation of the 3′ ends to the adapter ligation, with some modifications. The 49 amplicon libraries were then qualified to verify fragment size by checking the library size distribution via the 2100 Bioanalyzer (Agilent) using the High Sensitivity DNA chip. Quantification of the libraries was performed via qPCR using the Kapa library quantification kits for Illumina sequencing platforms (Kapa Biosystems). Prior to sequencing, final amplicon libraries were normalized to 2 nM and pooled. Finally, 600 μl of a 6 pM denatured pooled library with PhiX (30% final concentration) (Illumina, Inc.) was prepared, and a 300-bp paired-end sequencing was performed using the MiSeq reagent kit v3 (600 cycle) (Illumina, Inc.) according to the manufacturer's protocols. Sequencing was carried out at the Division of Analytical Bio-Medicine, Advanced Research Support Center (ADRES), Ehime University (Toon, Ehime Prefecture, Japan). Read data of the pooled libraries were demultiplexed using the on-board MiSEq Reporter software for the sequencing platform.

NGS data were analyzed using CLC Genomics Workbench and Galaxy (https://usegalaxy.org/) platform. The analysis included quality control of contigs, merging of forward and reverse contigs, adapter trimming, mapping into a reference (MNV S7-PP3), and creating the consensus sequences. Reads with fewer than 100 sequences were trimmed. The median of the score of reads of any base quality should be more than 20, measured using FastQC software on the Galaxy platform.

After the consensus sequences were generated, alignment between the consensus sequences in the same region was conducted using MEGA 7 software. In addition to the observation of the other SNPs, including minority sequences and their percentage in NGS reads, IGV (Integrative Genomics Viewer) software was used. A heat map representing the ratio of the percentage of SNPs to the percentage of nonmutated nucleotides in original population sequences in each cycle was determined using R software with a gene scale command available in the bioconductor package (https://www.bioconductor.org/). The *Z* score for the heat map was calculated by the following formula:

(1) $$Z=\frac{\left(x-\text{mean}\right)}{\text{SD}}$$

where *x* is the ratio of SNP percentages compared to that of the reference sequence in a certain cycle (cycles 1, 5, and 10). The mean value and standard deviation (SD) were calculated from *x* in all cycles separately per region. In addition, to characterize the evolutionary pattern of genetic divergence among populations with different treatments and treatment cycles, PCoA was conducted using CLC software (49). As the input distance matrix, Bray-Curtis dissimilarity was calculated based on the relative abundances (i.e., reads) of haplotypes in each population (50).

### Free chlorine susceptibility of plaque-purified clones.

In order to examine whether SNPs found in the capsid region are associated with the lower susceptibility of chlorine, a chlorine sensitivity test was carried out for clones isolated from both the control and chlorine-treated populations. The chlorine-treated and control populations of MNV S7-PP3 were inoculated to confluent RAW 264.7 cells in a 6-well plate with a multiplicity of infection (MOI) of 0.1. After 2 to 3 days of incubation, only clean and dispersed plaques were chosen for isolation. Plaques were picked using 1,000 μl of barrier tips cut by a sterilized cutter to obtain a clear, round shape. Six plaques were picked from each chlorine-treated and control population, mixed with DMEM, and incubated at 37°C for 90 min before being regrown in a new confluent of RAW 264.7 cells in 75-cm^2^ flasks containing 15 ml of DMEM. The first plaque-purified MNV clones were subjected to a second round of plaque purification. Each clone (12 in total, six each from the chlorine-treated and control populations) was confirmed to have the mutation at nt 7280. After the clones were isolated, they were stored in a −80°C freezer before conducting a free chlorine sensitivity test. The free chlorine sensitivity test was conducted using chlorine demand-free glassware, which was prepared by soaking overnight in a high concentration of free chlorine solution (>75 mg/liter). After soaking, the glassware was rinsed and baked at 120°C overnight and then thoroughly washed and sterilized by autoclave. The chlorine sensitivity test was conducted using 9:1 PBS and DMEM with FBS. Free chlorine concentration was measured three times with the U.S. EPA DPD method using DR 900 (Hach) equipment at time zero and after 5 min contact time. The chlorine decay time was determined according to the following formula:

(2) $$C_{t}=C_{0\;}\text{ exp}(-k\text{*}t)$$

where *C_t_* is the chlorine concentration at specific sampling times, *C*_0_ is the initial concentration of chlorine, k* is the chlorine decay rate (min^−1^), and *t* is the contact time (0, 0.5, 1, 2, 3, or 5 min). A preliminary chlorine decay experiment showed that the free chlorine (initial concentration, 2 ppm) in the PBS solution was fully consumed in 1 min. The chlorine decay rate (k*) calculated using equation 2 was 1.18 min^−1^. In the chlorine sensitivity assay, an X clone (from the control population) was paired with an A clone (from the chlorine-treated population), and nine pairs were selected randomly. Then, the log_10_ reduction ratio after free chlorine exposure with an initial concentration of 2 ppm and 1 min contact time was obtained 3 times for each pair. The free chlorine solution was made by mixing 50 μl of sodium hypochlorite stock solution (Wako, Osaka, Japan) with 100 ml PBS. After mixing by shaking for 10 to 15 s, 9 ml of the mixture was transferred to a small tube and 1 ml of clone suspension was added and mixed for 1 min. After 1 min, 10 μl of sodium thiosulphate solution 1% (vol/vol) was added to neutralize the chlorine. The original concentration of plaque-purified clones before and after chlorine treatment was measured by plaque assay.

### Replicative fitness of plaque-purified clones.

Replicative fitness was determined by comparing the growth rate between plaque-purified clones isolated from control and chlorine-treated populations. Low MOI (0.002 to 0.005) was applied for infection to the RAW 264.7 cells. Confluent RAW 264.7 cells in a 75-cm^2^ flask were rinsed twice with PBS and then infected with the targeting plaque-purified clones. After a 90-min incubation at 37°C, the flasks were rinsed with DMEM to remove the unattached virus particles. Virus samples were taken at 6, 12, 24, 36, and 48 h to obtain the growth curve (51). The growth rate was fitted from the lag phase of virus growth, and the following exponential fitting model was used (52):

(3) $$Y=e^{\mu t},\;\text{so ln}(Y)=\mu t$$

where *Y* is the virus yield, μ is the growth rate, and *t* is the cultivation time. To compare the relative replicative fitnesses of the different clones, the selection coefficient (*s*) was calculated using the following formula (30):

(4) $$s=1-\mu /\mu_{\text{WT}}$$

where μ and μ_WT_ are the log-phase growth rates of the evolved clones (plaque-purified clones isolated from the chlorine-treated population) and wild-type clones (plaque-purified clones isolated from the control population), respectively. An *s* value less than zero means that the evolved clones has a superior replicative fitness.

### Binding efficiency to cell surface.

A binding test was carried out according to a previous study with modifications (45). Briefly, RAW 264.7 cells were rinsed twice using PBS without calcium and magnesium [PBS (−)] to remove residual DMEM before the infection process. Virus was inoculated to the cells with a MOI of 0.1 and incubated at 4°C for 90 min to allow the attachment of virus particles, while preventing initiation of the replication cycle at low temperatures. Gentle rocking every 10 to 15 min was employed during the incubation period to prevent the cells from drying. RNA extraction was conducted as soon as incubation was complete. To remove unbound virus particles, the plates were rinsed three times with PBS (−). After rinsing, the cells were scraped using a cell scraper, and then a 150-μl mixture of cells and attached virus was subjected to viral RNA extraction with the QIAamp viral RNA kit (Qiagen). qPCR for MNV was employed to determine the copy numbers of viral RNA before and after the cell-binding experiment (53).

### Statistical analyses.

All statistical analyses were performed using R software version 3.3 (http://www.r-project.org). All data sets were assumed to not be normally distributed. To examine if the ratio of log_10_ reduction between clones isolated from the control population (X1, X2, X3) and the chlorine-treated population (A1, A2, A3) is less than 1 (null hypothesis), the Wilcoxon signed-rank test was employed. To determine if there was no statistical difference between the growth rate and the doubling time of each chlorine-treated and control clone, the Kruskal-Wallis test was conducted with a significance level of α = 0.05. To check the statistical difference between the selection coefficients (*s*) among clone combinations (A1 versus X1, A1 versus X2, A1 versus X3, A2 versus X1, A2 versus X2, A2 versus X3, A3 versus X1, A3 versus X2, and A3 versus X3), the Kruskal-Wallis test was performed. This test examined whether replicative fitness is statistically higher than 0. The ratio of bound viral particles to initial spiked virus between isolated clones (A1, A2, A3, X1, X2, X3) was tested using the Wilcoxon rank sum test.

## Supplementary Material

Supplemental material
