# Supplementary material for: Free-Chlorine Disinfection as a Selection Pressure on Norovirus
Source: Appl Environ Microbiol. 2018 Jun 18;84(13):e00244-18. doi: 10.1128/AEM.00244-18 (PMC6007107; doi:10.1128/AEM.00244-18)
Supplement: Supplemental material [file supp_84_13_e00244-18__index.html]

Supplemental material 

# Free-Chlorine Disinfection as a Selection Pressure on Norovirus

## Supplemental material

- Supplemental file 1 -

  Growth rate and doubling time for each clone isolated from the chlorine-treated population and the control population (Table S1); relative replicative fitness between clones isolated from the chlorine-treated population and the control population (Table S2); temperature profile of PCR for each of the seven regions from MNV ORF2 and ORF3 (Table S3); phylogenic tree of the chlorine-treated clones and the control clones derived from a murine norovirus S7 lineage (Fig. S1); ratio of MNV concentration in the supernatant after incubation at 4°C for 90 minutes to the initial MNV concentration (Fig. S2).

  PDF, 263K
