# Supplementary material for: Free-Chlorine Disinfection as a Selection Pressure on Norovirus
Source: Appl Environ Microbiol. 2018 Jun 18;84(13):e00244-18. doi: 10.1128/AEM.00244-18 (PMC6007107; doi:10.1128/AEM.00244-18)
Supplement: Supplemental material [file AEM.00244-18_zam013188592s1.pdf]

Table S1. Growth rate and doubling time for each clone isolated from the chlorine-treated population (A1, A2, A3) and the control population (X1, X2, X3)

Clone	Growth Rate (μ)/h	Doubling Time (H)
A1	0.76 ± 0.02	0.91 ± 0.02
A2	0.77 ± 0.02	0.90 ± 0.02
A3	0.80 ± 0.01	0.87 ± 0.01
X1	0.83 ± 0.01	0.86 ± 0.05
X2	0.82 ± 0.02	0.84 ± 0.03
X3	0.81 ± 0.01	0.85 ± 0.01

Table S2. Relative replicative fitness between clones isolated from the chlorine-treated population (A1, A3, A2) and the control population (X1, X2, X3)

Selection coefficient (s) value			
Clone	X1	X2	X3
A1	0.07	0.07	0.06
A2	0.06	0.07	0.05
A3	0.03	0.04	0.02

Table S3. Temperature profile of PCR for each seven regions from MNV ORF2 and ORF3.

Region	Temperature (°C)	Time	Cycle number
1	94	2 min	1
	94<br>66<br>72	30 s<br>45 s<br>45 s	20
	72	7 min	1
	4	∞	1
2	94	2 min	1
	94<br>68<br>72	30 s<br>45 s<br>45 s	20
	72	7 min	1
	4	∞	1
3	94	2 min	1
	94<br>67<br>72	30 s<br>45 s<br>45 s	20
	72	7 min	1
	4	∞	1
4	94	2 min	1
	94<br>66<br>72	30 s<br>45 s<br>45 s	20
	72	7 min	1
	4	∞	1
5	94	2 min	1
	94<br>70<br>72	30 s<br>45 s<br>45 s	20
	72	7 min	1
	4	∞	1
6	94	2 min	1
	94<br>64<br>72	30 s<br>45 s<br>45 s	20
	72	7 min	1
	4	∞	1
7	94	2 min	1
	94<br>64<br>72	30 s<br>45 s<br>45 s	20
	72	7 min	1
	4	∞	1

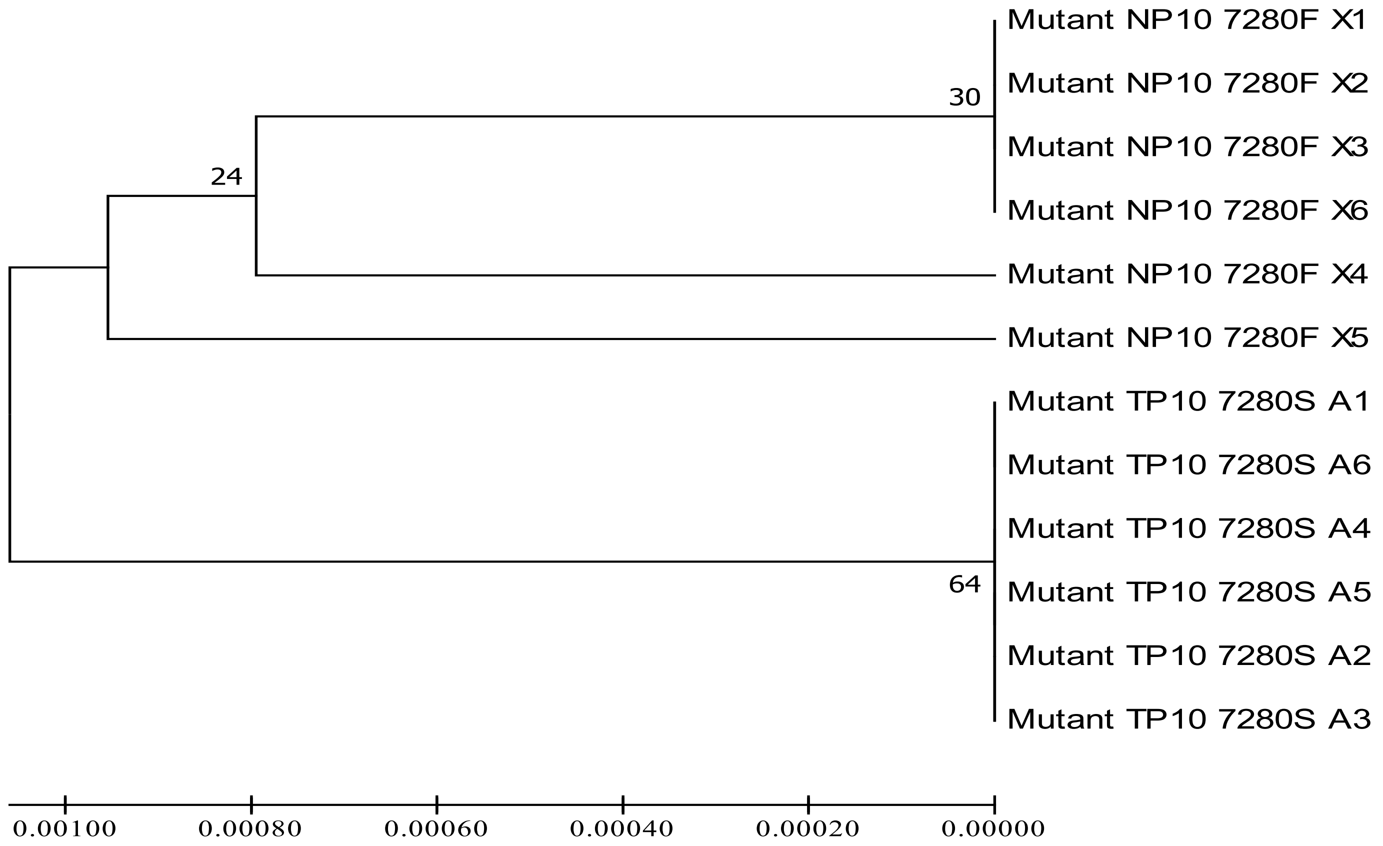

Fig. S1. A phylogenic tree of the chlorine-tretaed clones (A1, A2,A3, A4, A5, A6) and the control clones (X1, X2, X3, X4, X5, X6) derived from a murine norovirus S7 lineage. Forward and reverse SANGER sequences for each strains was merged using ExPasy website. Merged sequences were aligned by MUSCLE and a phylogenetic tree was generated using bootsrap method with 5000 replications in MEGA 7 software. Evolutionary distances were compute using Tamura-Nei model, and evolutionary history was inferred usingUPGMA method.

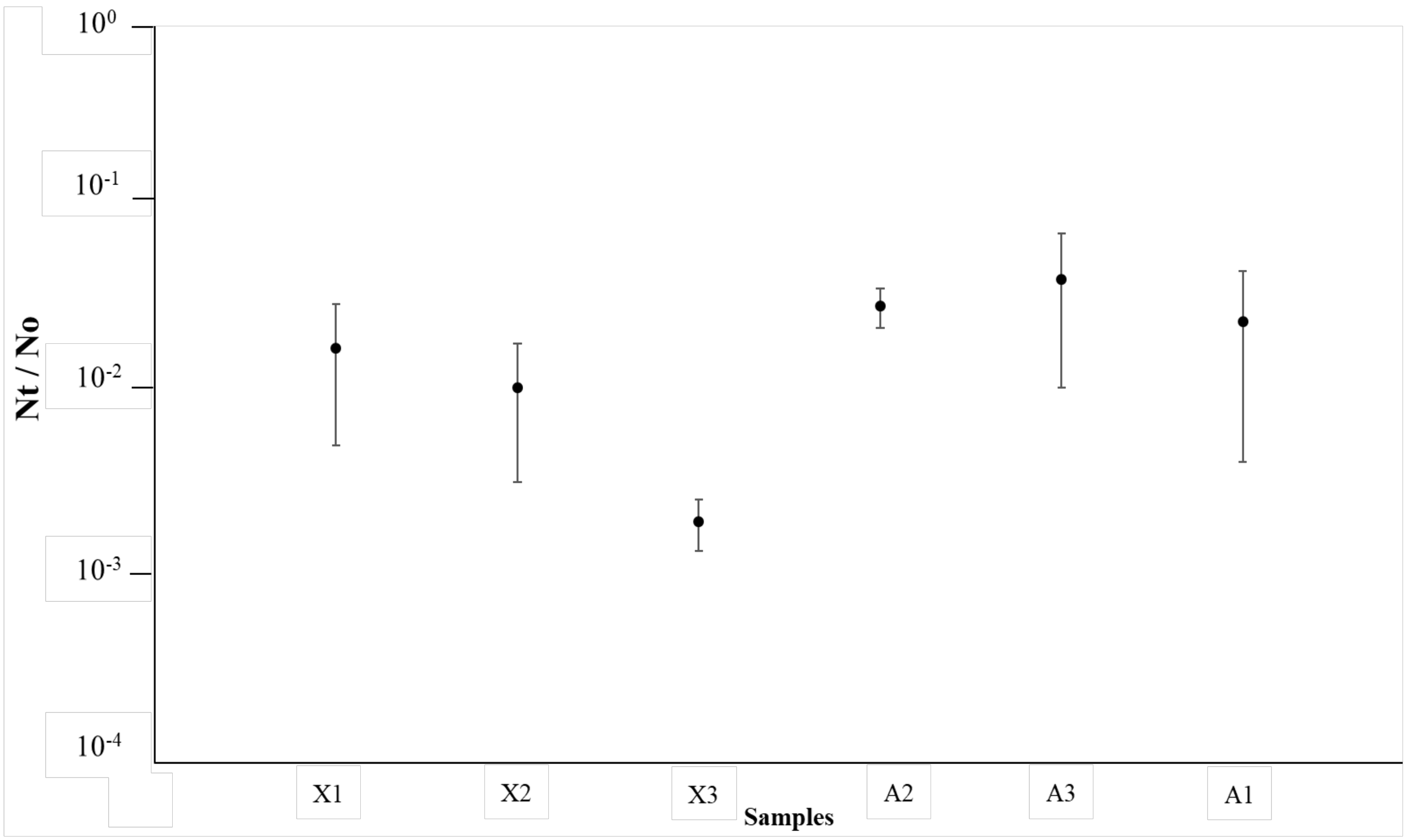

Fig. S2. The ratio of MNV concentration in the supernatant after incubation at 4 ˚C for 90 minutes (N_t) to the initial MNV concentration (N_0).
